# World Hepatitis Day — July 28, 2018

**DOI:** 10.15585/mmwr.mm6728a1

**Published:** 2018-07-20

World Hepatitis Day is commemorated each year on July 28 with the goal of promoting awareness and inspiring action to prevent and treat viral hepatitis. The World Health Organization’s (WHO’s) theme of this year’s World Hepatitis Day is “Test. Treat. Hepatitis” to underscore the urgent need to scale up testing and treatment activities.

WHO estimated that globally in 2015, approximately 325 million persons were infected with hepatitis B virus (HBV) or hepatitis C virus (*1*). Among the estimated 257 million persons infected with HBV in 2015, nearly 900,000 died, primarily as a result of complications of cirrhosis and hepatocellular carcinoma (*1*). In 2016, the World Health Assembly endorsed viral hepatitis elimination goals set by WHO, defined as a global reduction of 90% in incidence of and 65% in mortality from hepatitis B and hepatitis C by 2030 (*1*).

This issue of *MMWR* features a report on progress toward access to hepatitis B treatment worldwide. Overall, hepatitis B treatment coverage is low among countries in all income strata. Increased awareness of, access to, and availability of affordable diagnostics, and training of health care providers might increase access to treatment. Additional information and resources are available at https://www.cdc.gov/hepatitis.
